# The Importance of the Mineral Substrate of the Biofilm in the Process of Low-Temperature Removal of Nitrogen Compounds from Wastewater

**DOI:** 10.3390/ma16237417

**Published:** 2023-11-29

**Authors:** Anna Maria Anielak, Michał Polus, Helena Diakun, Izabela Radomska-Kreft

**Affiliations:** 1 Faculty of Environmental Engineering and Energy, Cracow University of Technology, Warszawska 24, 31-155 Krakow, Poland; 2 Municipal Sewage Treatment Plant in Człuchów Przedsiębiorstwo Komunalne Spółka z o.o., ul. Sobieskiego, 11, 77-300 Człuchów, Poland; helena.diakun@wp.pl (H.D.); radomskakreftiza@gmail.com (I.R.-K.)

**Keywords:** nitrification, denitrification, SBR, biofilm, ammonia-oxidizing archaea, ammonia-oxidizing bacteria, simultaneous nitrification and denitrification, nitrogen compounds

## Abstract

This study researched the use of biofilms to remove nitrogen compounds from municipal sewages at low temperatures, especially in winter. An aluminosilicate substrate was used to create a biofilm, which has an affinity for ammonium ions. The selection of biofilm-forming microorganisms has been shown to occur on aluminosilicate. This substrate is mainly inhabited by microorganisms that remove nitrogen compounds. As a result, microorganisms protected against external factors in the biofilm effectively remove nitrogen compounds. The TN content in sewage treated at a temperature of 10 °C was of a 4 mg/L order and was 3–5 times lower than in the reference system (classical conditions). This process involves shortened nitrification/denitrification such as Anammox. As a result of a given process, CO_2_ emissions were reduced and much smaller amounts of NO_x_ were produced, positively impacting the ongoing climate changes. Microbiological DNA/RNA tests have shown that the biofilm is primarily composed of archaea and bacteria that remove nitrogen compounds, including those that oxidize ammonia.

## 1. Introduction

The task of every sewage treatment plant is to remove organic and inorganic pollutants. Important pollutants include nutrients that contribute to the eutrophication of surface streams and can be toxic in high concentrations (nitrogen compounds) [1].

Removing nitrogen compounds from sewage is an energy-intensive process, and two-stage nitrification requires aeration, which is the most expensive process. Aeration in conventional wastewater treatment plants accounts for 25–60% of the overall costs accumulated during the wastewater treatment process. Usually, good results are achieved using large-scale technologies. In a conventional nitrification/denitrification process, a COD/N ratio > 3.5 is required to achieve full nitrogen removal efficiency. If the quotient is lower than 3.5, Anammox-based methods should be used to obtain effectively higher nitrogen removal rates and economic feasibility of treatment [2].

The biological removal of nitrogen compounds is a process with effectiveness that depends on many factors, including oxygen content, organic substances, amounts of recirculated humus acids from digested sludge [3], and others, of which the temperature of the sewage is important [1,4,5]. The values of this parameter fluctuate throughout the day and year. The nitrification process is stable at temperatures of 28–35 °C. Significant variability in process efficiency begins below 22 °C. This process is clearly inhibited in the temperature range of 0–10 °C [6,7,8]. Research shows that lowering sewage temperatures helps reduce the number of bacteria and archaea. Similar dependencies were obtained for soil, where humidity and temperature affect the rate of ammonification and nitrification [9,10]. Previous research indicates that ammonia-oxidizing bacteria (AOB) dominate in the soil at low temperatures, and ammonia-oxidizing archaea (AOA) dominate at high temperatures [11]. At a temperature of 15 °C, for example, in Arctic soils, AOA quantitatively dominates (AOA outnumbers AOB by 1–3 orders of magnitude in soils where both were detected); even with effective nitrification observed, the amount of AOB, expressed as the detectable number of copies of the ammonia monooxygenase gene (amoA), is undetectable using molecular methods (PCR) [12]. The authors of [11] believe that the effect of temperature on the nitrification process depends on the AOA to AOB ratio. The nitrification process mainly involves bacteria that oxidize ammonia and nitrite (NOB). The authors of Kruglova et al. [7] state that there are described species of AOB and NOB adapted to low temperatures [13,14]. In [15], a medium DO concentration (0.7 mg O_2_/L) was used at low and moderate temperatures (10–16 °C), thus obtaining appropriate AOB activity in relation to NOB with low energy consumption. It is generally assumed that the optimal temperature for activated sludge is 20–40 °C. Gonzalez-Martines et al. [16,17] believe that the diversity of bacteria and archaea in activated sludge is lower at lower sewage temperatures.

An example is Collings et al.’s proposal [10] denitrifying down-flow hanging sponge (DDHS) reactors about efficiency >58%. However, even in this solution, there was a significant reduction in the efficiency of TN removal at 13 °C and a complete reduction at 6 °C. However, there was effective nitrification of ammonium nitrogen of >90%. Kruglova et al. [7] believe that with sharp temperature fluctuations, the efficiency of TN removal can be increased by extending the sludge retention time from 14 to 100 days. Experiments by Ye et al. [18] showed that the bacterium *Pseudomonas putida* Y-12 can be used to remove nitrites. The tests were conducted on synthetic solutions with dissolved nitrogen salts (−5, +5, and +3). The results indicate that the Y-12 strain can be used at low temperatures for heterotrophic nitrification and aerobic denitrification. Zhong et al. [19] isolated bacteria *A. jonsonii* strain F and *A. bereziniae* strain H, which can efficiently remove NH_4_-N at low temperatures (15 °C) without accumulating NO_3_-N/NO_2_-N. A putative plasmid containing an antibiotic-resistant gene was detected in the F strain. Studies conducted at five full-scale Finnish sewage treatment plants showed the influence of the temperature and composition of sewage on the treatment process efficiency. In the activated sludge, a low number of AOB < 0.25% of the total population, NOB < 0.35%, and a high number of orders *Cytophagales*, *Micrococcales*, and Candidatus *Nitrotoga arctica*, which oxidize nitrites at low temperatures, were found in the activated sludge [7].

Biological treatment can be carried out with activated sludge or biofilm, which is a structural aggregate formed by microorganisms [20,21]. Biofilm microorganisms are protected against chemical and physical stress and shear forces, have the ability to survive in very difficult conditions, and are resistant to immunological clearance and antibiotic therapy. Studies have shown that biofilms are an economically viable option to replace activated sludge. Additionally, biofilm usage is a basic method for microorganisms to prevent external stress. Biofilms generate extracellular polymeric substances (EPS), SOS, and quorum sensing. These substances protect biofilms from chemical destruction. Surface colonization can also concentrate nutrients. In other words, biofilm bacteria can adjust the biofilm structure via mass transfer to enter a local growth mode under capricious environmental conditions, which ensures flexibility to adapt quickly [22].

Therefore, they are a good solution for treating wastewater that contains substances that are toxic to biological processes. It is believed that this approach solves some disadvantages of sludge processes that require expensive disposal of sewage sludge generated in the wastewater treatment process [23,24,25].

However, this method has its drawbacks, with the main problem being the long startup time of biofilm technology. Some studies have shown that biofilm ensures simultaneous nitrification and denitrification (SND) [22,26]. This process involves various microorganisms responsible for denitrification and nitrification, which interact synergistically. Nitrifiers are located in the outer zone of the biofilm. SND eliminates all the shortcomings of traditional nitrification and heterotrophic denitrification and is characterized by high levels of nitrogen removal.

Therefore, based on the experience of various authors and our own research [27], it was decided to investigate the influence of the mineral substrate on the formation of biofilm and the removal of nitrogen compounds. This study aimed to investigate the influence of the mineral substrate and biofilm on the wastewater treatment process at low temperatures when the nitrification process is inhibited. Our research assumed that a mineral deposit with an affinity for nitrogen compounds creates good conditions for developing microorganisms and increases the effectiveness of nitrification and denitrification processes.

## 2. Materials and Methods

### 2.1. Place of Research

**Figure 1:**
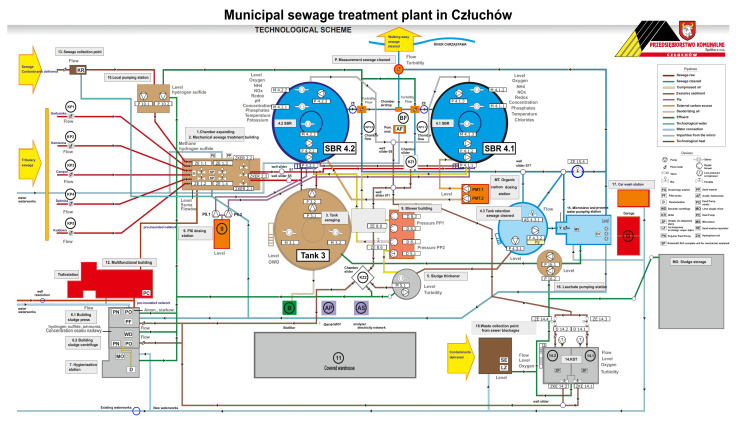
Municipal sewage treatment plant in Człuchów. Technological scheme.

This research was carried out at the municipal sewage treatment plant in Człuchów. The municipal sewage treatment plant in Człuchów is a mechanical and biological sewage treatment plant of the SBR type, designed for 26,130 inhabitants and 2371 m^3^/d. Sewage is delivered to the sewage treatment plant from the area of the urban and rural communes of Człuchów via sanitary sewage systems and sewage disposal trucks. The diagram of the sewage treatment plant is shown in Figure 1.

Raw sewage via chamber expansion (1) flows to the mechanical sewage treatment building, where it is separated into two independently operating screen sand traps (2), where screenings, sand, and fats are removed. Mechanically pre-treated sewage gravitationally flows through the separation chamber to the averaging tank (3) from where it is pumped further to two biological reactors of the SBR type (4.1) and (4.2). The phases of reactor operation, aerobic and anaerobic conditions, and filling and decanting of sewage are shown later in the workBiological phosphorus removal is supported using a PIX coagulant dosed into SBR reactors from the PIX dosing station (9). In addition, sludge management is conducted.

### 2.2. Mineral Substrate of the Biofilm

**Figure 2:**
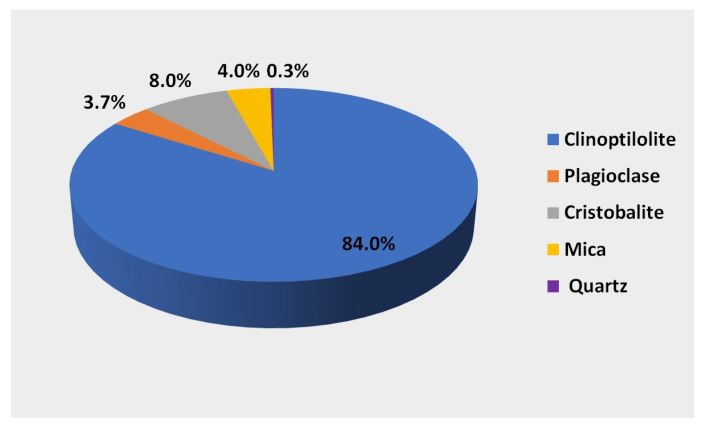
Mineral composition of the substrate used in the tests.

A natural mineral formed in natural conditions as a result of changes occurring in volcanic ash under the influence of high pressure and temperature was used to create the biofilm. The mineral used is characterized by a high clinoptilolite content of 84%. It is a hydrated alkali metal aluminosilicate composed of aluminum-AlO_4_ silicon SiO_4_ tetrahedra connected at the corners with common oxygen atoms. Aluminosilicates have a spatially cross-linked structure with regular three-dimensional pores forming tunnels. The unevenly distributed positive charge (Al^3+^) (Si^4+^) in the tetrahedra does not compensate for the negative charge of oxygen (O_4_^2-^), which generates an excess of negative charge and forms an electronegative structure. The excess negative charge and the porous tunnel structure give the mineral unique sorption, ion exchange, and catalytic properties. The mineral composition [28] of the zeolite used in the research is shown in Figure 2. In addition to clinoptilolite, it contains 8% cristobalite, 4% mica, 3.7% plagioclase, and 0.2–0.3% quartz.

Qualitative analysis indicates that its structure is dominated by silicon SiO_2_, the content of which is 60–72%, and aluminum (Al_2_O_3_) in the amount of 11.5–14%. The remaining elements are calcium (CaO 2.7–5.3%), potassium (K_2_O 2.2–3.4%), iron (Fe_2_O_3_ 0.7–1.9%), magnesium (0.6–1.2%), sodium, and titanium [28]. Due to the excess of negative charge in the structure, this mineral has ion exchange capabilities that depend on the ion being exchanged. The mineral has the greatest affinity for ammonium ions >0.70 mol/kg, calcium ions 0.64–0.98 mol/kg, and potassium ions 0.22–0.45 mol/kg.

### 2.3. Characteristics of Raw Sewage Sent to the Sewage Treatment Plant in Człuchów

**Figure 3:**
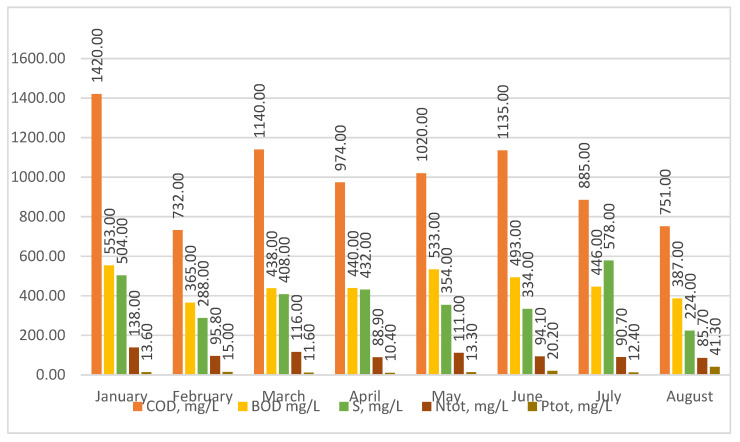
Physical and chemical characteristics of raw sewage treated at the sewage treatment plant in Człuchów.

The analysis of the test results shows (Figure 3) that the sewage treated at the Człuchów sewage treatment plant is typical for social and domestic sewage. The content of COD in raw sewage, during the period studied, ranged from 1420 to 732 mg/L, BOD from 553 to 365 mg/L, sulfur from 578 to 224 mg/L, TN from 138 to 85.7 mg/L, and TP from 41.3 to 10.4 mg/L. No heavy industry or plants generate toxic substances in Człuchów. The food industry is mainly developing in the region. Therefore, sewage directed to treatment plants should be treated as easily purified using the activated sludge method in the Sequential Beach Reactors (SBRs) system.

### 2.4. Process Research Methodology

**Figure 4:**
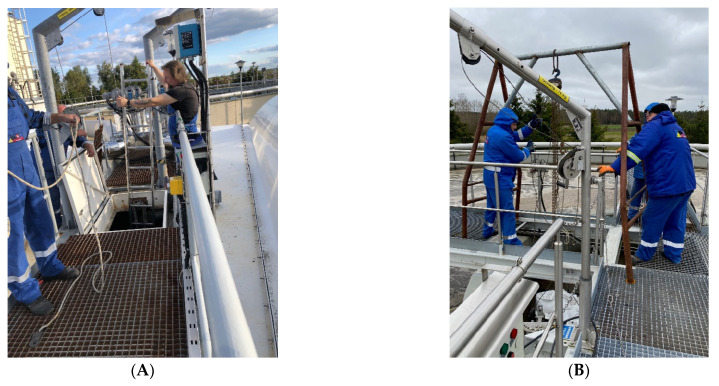
Installation of the mineral substrate (**A**) in the retention tank (tank 3) and (**B**) in the central chamber of the SBR 4.1 reactor.

This research was conducted on two systems. In the first one, lasting from September 2022 to December 2022 (76 days), 1 ton of mineral substrate was placed (Figure 4A) at the bottom of retention tank (3 Tank), to which raw sewage flowed. Then, from this tank, it was alternately distributed to one and a second SBR reactor. Samples of treated wastewater were collected separately from the outlet of each reactor and analyzed. The results obtained were compared with those obtained in 2021 in the same period of the year.

In the second system, the tests involved placing a biological substrate (Figure 4B) at the bottom of the central part of the SBR 4.1 reactor. There is a continuous recirculation of sewage in this place, which ensures good mixing and contact of microorganisms with the mineral substrate. Our research was conducted in the months from January to June 2023. The system of SBR reactors and the retention tank are shown in the diagram of the treatment plant in Figure 1. The reactor in which the mineral substrate SBR 4.1 was placed is shown in the diagram on the right. The second SBR 4.2 reactor is shown in the diagram on the left. Figure 4 shows the installation of the mineral substrate (A) in the retention tank (tank 3) and (B) in the central chamber of the SBR 4.1 reactor, which established the reference level for the results of the qualitative analysis conducted for sewage treated in the SBR 4.1 and 4.2 reactors.

To assess the efficiency of wastewater treatment in a classic system and a system with a biological substrate, the following indicators were determined for the treated wastewater in the tested systems: BOD, COD, pH, temperature, suspended solids (TSS), total phosphorus (TP), N-NH_4_, N-NO_2_, N-NO_3_, and total nitrogen (TN). Additionally, the following were measured 24 h a day using probes and electrodes with continuous operation at the sewage treatment plant: temperature, oxygen concentration, suspended solids, TP, N-NH_4_, pH, and NO_x_.

Additionally, other parameters were measured using probes continuously operating around the clock. The measurement installation consists of an Endress + Hauser measurement probe: ISEmax CAS40D (nitrogen compounds, chlorides, potassium), COS61D (dissolved oxygen measurement, temperature), CPS12D (redox), TURBIMAX CUS51D (concentration measurement), FRM 20 (concentration measurement), CA80PH phosphate analyzer, Hach measurement probes: AS-ISE sc type. LXV440.60.00001 (nitrogen compounds, chlorides, pH, potassium). All measurement data from the above-mentioned measurement systems are transferred to the SCADA Adroit visualization program. The author of the visualization system is AT Control System Sp. z o. o. Gdańsk. DNA/RNA analyses were performed to detect the presence of bacteria and archaea for the mineral substrate with biofilm.

### 2.5. Microbiological Testing

The attempt to assess the quantitative relationship between ammonia-oxidizing bacteria (AOB) and ammonia-oxidizing archaea (AOA) results from the different biological characteristics of these microorganisms and their life strategies. These differences are also visible between the development of microorganisms in the natural environment and under experimental conditions. As a rule, AOB dominate in the microbial community due to their rapid growth. This applies especially to situations where the oxygen concentration is relatively high, the NH_4_^+^ concentration is also high, and the pH is slightly alkaline. A high NH_4_^+^ concentration is usually poorly tolerated by AOA, and the deficiency of available carbon prevents the heterotrophic (or mixotrophic) lifestyle of AOA, allowing only slow autotrophic development [29]. In turn, in anoxic conditions, when the availability of NH_4_^+^ is limited and the pH goes beyond the range of 6–8, the growth of AOB should be strongly limited and their share in the community reduced. Consequently, this research was conducted to assess the proportion of bacteria to archaea in the biocoenosis developing in the sediment. Semi-quantitative tests performed in our model allow us to conclude that although the conditions in the reactors favor the development of archaea, they do not seem to be involved in the transformation of NH_4_^+^.

The material for genetic analysis was a mineral, porous substrate with biofilm, coming from a biological batch reactor. The presence of ammonia-oxidizing bacteria (AOB) and ammonia-oxidizing archaea (AOA) was assessed in this material. A unique enzyme catalyzing the oxidation of ammonia was used to detect AOA. For this purpose, primers designed to target the α-subunit of ammonium monooxygenase were applied. The presence of all archaea was estimated based on the presence of the 16S rRNA gene, which was also used for genera identification. Similarly, ammonia-oxidizing bacteria were detected using primers directed at the 16S rRNA sequence typical for AOB and the ammonium oxidase gene. Primer sequences are listed in Table 1.

Briefly, a biofilm gathered from a substrate taken from a bioreactor was used to isolate total DNA. Approximately 10 g of the substrate was suspended in 50 mL PBS and shaken to separate the biofilm. The collected suspension was concentrated by centrifugation (5000× *g*, 5 min). The collected pellet was suspended in 150 µL of a cooled sterile 0.9% NaCl solution and DNA isolation and purification were performed using a column kit from EURx (Gdańsk, Poland) designed to work with environmental samples such as sewage sludge or soil, according to the manufacturer’s protocol. Purified DNA was eluted from the columns with 50 µL of nuclease-free water and stored frozen at −20 °C.

The PCR reaction was performed on a Mastercycler Nexus Gradient thermal cycler (Eppendorf, Hamburg, Germany). Then, 1 µL of DNA purified in the previous step was used for the reaction, and 2.5 units of recombinant, thermostable DNA polymerase (Biotools, Madrid, Spain) were used for each reaction. The thermal profile of the reaction included the following: denaturation at 94 °C/45 s, annealing at 53 °C/45 s, and extension at 72 °C/60 s for a total of 35 cycles. PCR products were separated on an agarose gel and stained with SYBR Gold (Thermo Fisher Scientific, Waltham, MA, USA). The semi-quantitative evaluation was performed using a series of decimal dilutions of the tested DNA for the PCR reaction.

## 3. Results

### 3.1. Physical and Chemical Characteristics

The results of the tests conducted according to the first system are presented in Table 2. The comparison of these results with those obtained in the same period at the sewage treatment plant a year earlier did not show any influence of the substrate on the efficiency of sewage treatment, and no trends in changes in the quality of treated sewage were found throughout the entire observation period (76 days). Large standard deviations indicated significant variation in the study results. Therefore, further observations were discontinued.

**Figure 5:**
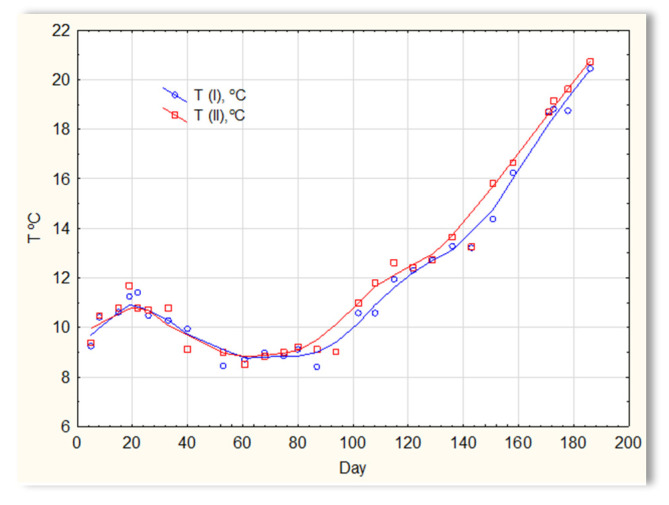
The sewage temperature in reactors T(I) in the SBR 4.1 reactor and T(II) in the SBR 4.2 reactor.

**Figure 6:**
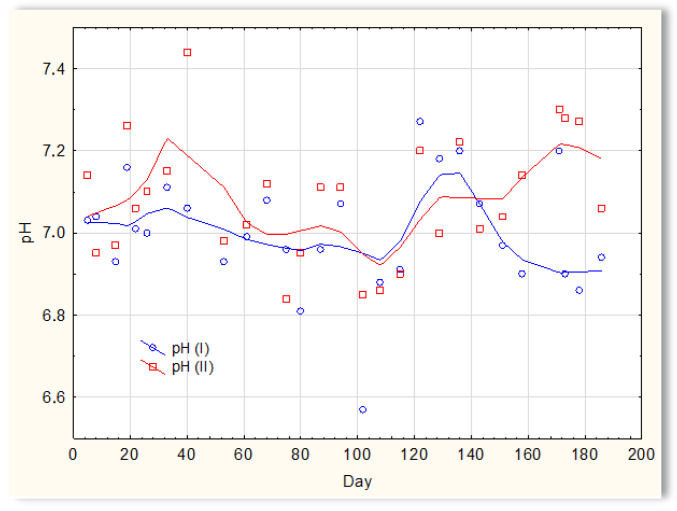
pH of the sewage treated in the SBR 4.1 pH(I) and SBR 4.2 pH(II) reactor.

The second series of tests was carried out in winter, which resulted in a significant reduction in the temperature of the sewage (Figure 5). The sewage temperatures in SBR 4.1 and 4.2 were at the level of 10 °C on the first days of the tests. On the 20th day of measurements, it increased to 11–12 °C; then, on the 40th day, it dropped below 10 °C and remained at 8–9 °C for approximately 50 days. From day 100, a slow, linear increase in temperature was observed, and it reached 21 °C only on the last day of research (day 186). Therefore, this research was conducted in a relatively difficult period for sewage treatment plants [1,2]. For the biological wastewater treatment process, the recommended temperature is 18–22 °C. Many years of experience in the operation of biological sewage treatment plants indicate that lowering the temperature to 15 °C results in a change in the efficiency of the sewage treatment process. A temperature drop to 10 °C significantly reduces the effectiveness of nitrification and contributes to an increase in the TN concentration in the outflow. Lower temperatures may inhibit nitrification. The pH of sewage in SBR 4.1 and 4.2 (Figure 6) remained constant throughout the entire research period and amounted to approximately 7 pH. Little variation occurred after day 150. During this time, a slight increase in the pH of sewage treated in reactor 4.2 was observed to be approximately 7.2–7.3, and a decrease in the pH of sewage treated in reactor 4.1 to approximately 6.9.

**Figure 7:**
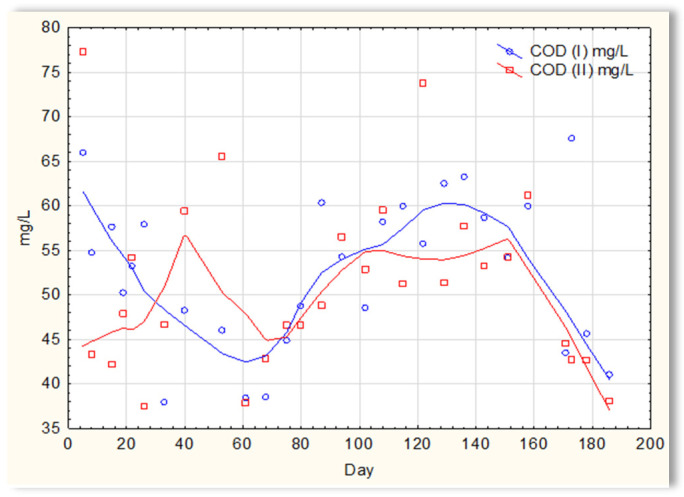
COD of sewage treated in the SBR 4.1 and SBR 4.2 reactor.

**Figure 8:**
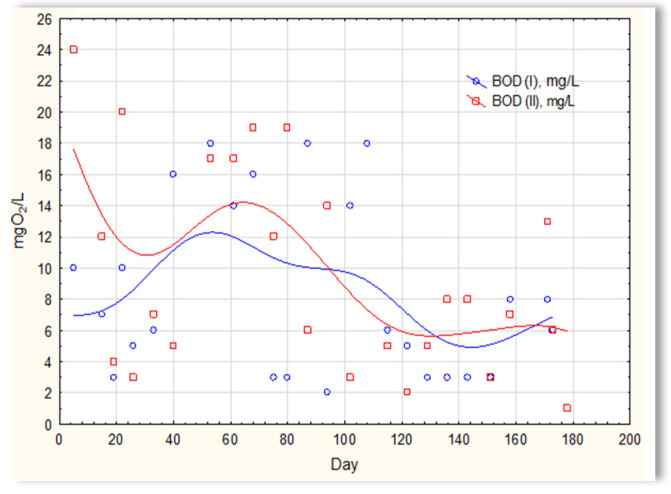
BOD of sewage treated in the SBR 4.1 and SBR 4.2 reactor.

**Figure 9:**
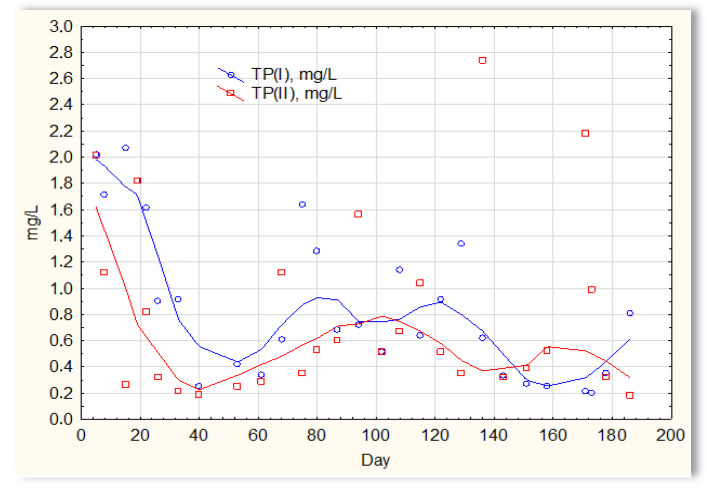
Concentration of phosphorus compounds in sewage treated in the SBR 4.1 (TP(I)) and SBR 4.2 reactor (TP(II)).

**Figure 10:**
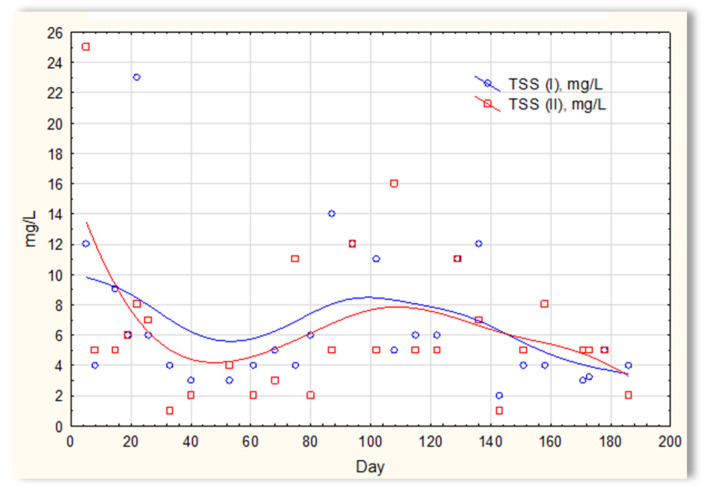
Concentration of suspended solids in sewage treated in the SBR 4.1 (TSS(I)) and SBR 4.2 reactor (TSS(II)).

The analysis of the test results presented in Figure 7 indicates that during 180 days of observation, there were no significant differences in COD values in sewage treated in the SBR 4.1 and 4.2 reactors. Similar relationships were obtained for BOD (Figure 8), i.e., the differences in the values of this parameter in the sewage treated in the SBR 4.1 and 4.2 reactors remained at a constant level, and there was no constant tendency in the discrepancies in the numerical values. Small differences were observed in the concentration of phosphorus compounds (Figure 9) and suspended (Figure 10). Sewage treated in the SBR 4.1 reactor, in which a mineral substrate was installed, can be assumed to have an increased TP concentration from day 1 to 150, on day 20 by 0.8 mg/L, and on the remaining days by about 0.2–0, 3 mg/L. From day 150, divergent pH values of treated sewage and a decrease in COD values in treated sewage in both reactors have been observed.

**Figure 11:**
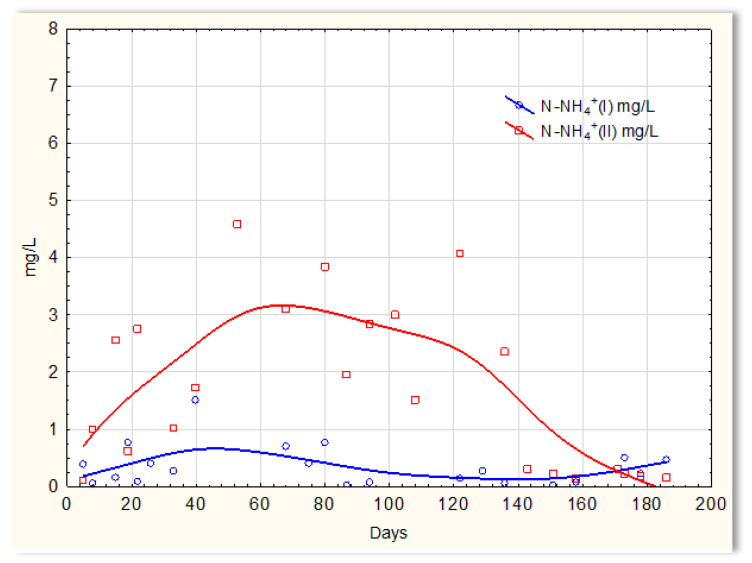
N-NH_4_ concentration values in sewage treated in the SBR 4.1 reactor (N-NH_4_ (I)) and in the SBR 4.2 reactor (N-NH_4_ (II)).

Completely different relationships were obtained for nitrogen compounds. The graphs indicate that the sewage temperature in the SBR 4.2 reactor significantly impacts the efficiency of ammonium ions removal from it (Figure 11), and there was a large variation in the results regarding the efficiency of nitrification in reactor 4.1, where a mineral substrate with biofilm was installed. The greatest variation occurred on days 40 to approximately 138 and was of the order of several mg/L of ammonium nitrogen. Exactly this difference in the efficiency of ammonium ion removal occurred when the wastewater temperature was <14 °C. Particularly difficult conditions for the nitrification process are observed at temperatures <10 °C. The temperature increase to 12 °C (day 120) did not change the observed relationship; the concentration of ammonium ions in the sewage treated in the SBR 4.2 reactor on day 121 was 4 mg/L. Only an increase in temperature to 14 °C improved the effectiveness of nitrification. The concentration of ammonium ions in treated sewage dropped to 0.2–0.1 mg/L. However, in the presence of a mineral substrate with biofilm, the removal of ammonium ions was significant, and the remaining amounts in treated sewage were at the level of 0.1–0.2 mg/L. Occasionally, the concentration of ammonium ions was higher but always below 1 mg/L.

**Figure 12:**
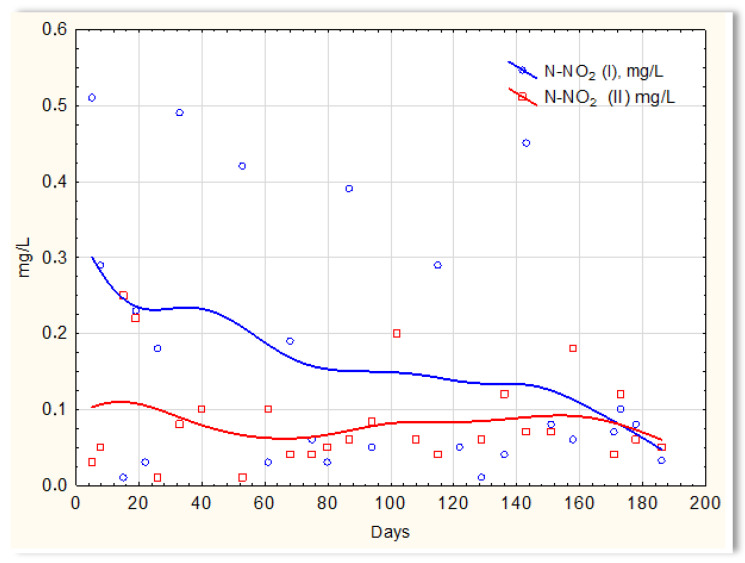
N-NO_2_ concentration values in sewage treated in the SBR 4.1 reactor (N-NO_2_ (I)) and in the SBR 4.2 reactor (N-NO_2_ (II)).

**Figure 13:**
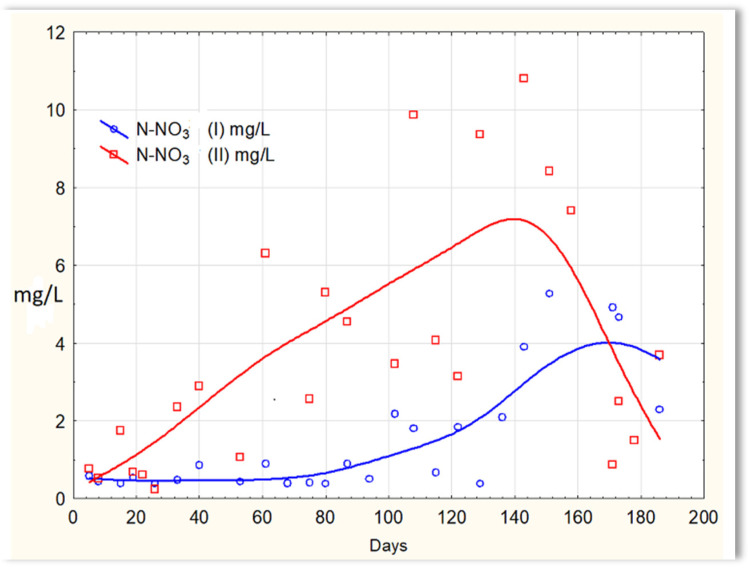
N-NO_3_ concentration values in sewage treated in the SBR 4.1 reactor (N-NO_3_ (I)) and in the SBR 4.2 reactor (N-NO_3_ (II)).

Inverse relationships were obtained for the N-NO_2_ content in sewage treated in the SBR 4.1 and 4.2 reactors. The concentration of this form of nitrogen was higher in sewage treated in the SBR 4.1 reactor than in SBR 4.2 (Figure 12). The existing dependencies can be explained by the N-NO_3_ content in treated sewage (Figure 13). Until the 170th day of observation, the N-NO_3_ concentration was higher in sewage treated in the SBR 4.2 reactor. The largest amounts of N-NO_3_ were obtained on the 140th day of observation. However, the amounts of N-NO_3_ for the SBR 4.1 reactor up to day 140 were <2 mg/L. The obtained test results indicate that in the SBR 4.1 reactor, the nitrogen compound removal process consists of two stages: in the first, the partial oxidation of ammonium nitrogen to N-NO_2_ takes place; in the second, N-NH_4_ and N-NO_2_ are directly converted into diatomic nitrogen (N_2_). Nitrogen removal is anaerobic.

This is the so-called autotrophic denitrification, in which, after the oxidation of ammonium nitrogen to nitrates (III), there is a reduction in nitrates (III) to NO_X_ and molecular nitrogen N_2_:

NH_3_ → NO_2_^−^ → NO → N_2_O → N_2_

This sentence does not contain information that the process takes place in aerobic conditions. The degree of oxidation of nitrogen increases from −5 to +3 (in other words, nitrogen is oxidized). This process occurs in anaerobic conditions. Many authors believe that biofilms consist of aerobic (external) and anaerobic (internal) layers, especially when formed in SBR, where alternating oxygen and anaerobic conditions occur. Therefore, oxygen involvement cannot be ruled out, but this requires separate and detailed tests.

Bacteria in this process participate in autotrophic respiration, and there is no need for an available source of organic carbon in the wastewater. Less need to oxidize ammonia also results in energy savings due to less oxygen demand. The process is analogous to Anammox [30,31,32].

In the SBR 4.2 reactor, where there was no mineral substrate with biofilm, there were only planktonic bacteria. Two-stage nitrification to N-NO_3_ takes place, and then denitrification to molecular nitrogen (N_2_). Therefore, the concentration of N-NO_3_ is higher in the sewage treated in SBR 4.2 than in the SBR 4.1 reactor. Bacteria participating in the nitrification process (*Nitrosomonas* and *Nitrobacter*) are chemolithoautotrophs that use ammonium and nitrate nitrogen (III) as electron donors for their synthesis processes. Nitrogen removal in aerobic conditions is possible either by using bacteria capable of simultaneous aerobic and nitrate respiration or by carrying out simultaneous nitrification and denitrification in a single apparatus. The latter process is carried out by separating oxygen and anoxic zones in the device. In the SBR 4.2 reactor, there are aerobic, anoxic, and anaerobic conditions, favoring two-stage nitrification and denitrification to N_2_.

However, there is more N-NO_2_ in the sewage treated in reactor 4.1, which is the final product of nitrification, and one-stage nitrification to N-NO_2_ takes place.

**Figure 14:**
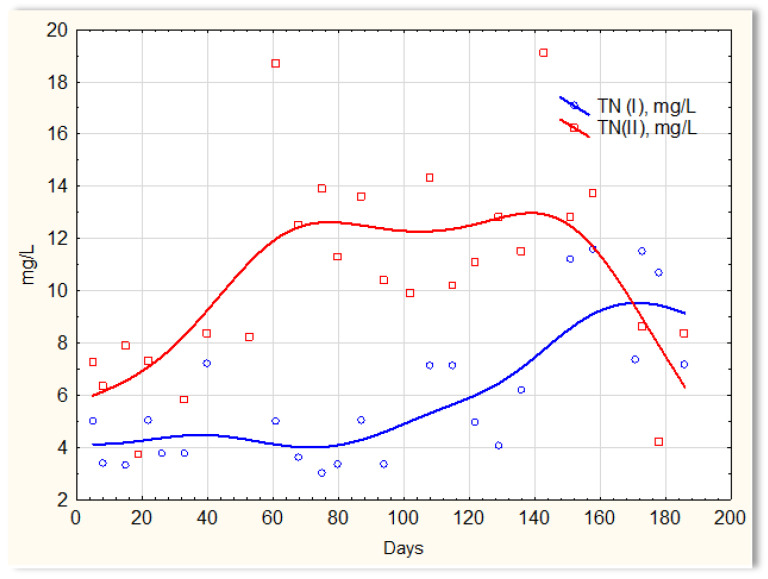
TN content in sewage treated in the SBR 4.1 (TN I) and SBR 4.2 (TN II) reactor.

The activities of the biofilm are shown in Figure 14, which shows a clear difference in the efficiency of removing nitrogen compounds in the presence of and without the biofilm. Similar relationships were obtained in previous studies in which the mineral substrate was used in the form of a loose solid phase, dosed into sewage, and treated in SBR reactors [27]. Sewage treated in the SBR 4.1 reactor (with biofilm) had a TN of approximately 4 mg/L until day 120, then it increased to approximately 6 mg/L on about day 120. However, the TN concentration in the SBR 4.2 reactor (Figure 14) was at the level of 12–14 mg/L, and in individual cases, it was even 19 mg/L. The achieved efficiency of nitrogen compound removal results from the conditions in which the biofilm is formed, which affects the type of microorganisms developing in it participating in the process of anaerobic nitrogen compound removal. There are no data in the literature regarding the possibility of simultaneous nitrification and denitrification in homogeneous conditions, i.e., without separated aerobic and anoxic zones. It can, therefore, be assumed that the formed biofilm has an internal anaerobic layer (inside the substrate structure) and an external aerobic layer that is in direct contact with the sewage. First of all, the substrate shows a high sorption of ammonium ions and catalyzes the process of anaerobic denitrification. Biofilm has a beneficial effect on only the processes of nitrification and denitrification. The remaining contaminants are not removed via the biofilm microorganisms. Microorganisms that aggregate in the biofilm are protected against chemical factors and are resistant to external stress. Biofilm cells are many times more resistant to low temperatures than bacteria under classical conditions (SBR 4.2 conditions). The effective removal of nitrogen compounds indicates that specific microorganisms capable of nitrifying and denitrifying nitrogen compounds aggregate in the biofilm.

**Figure 15:**
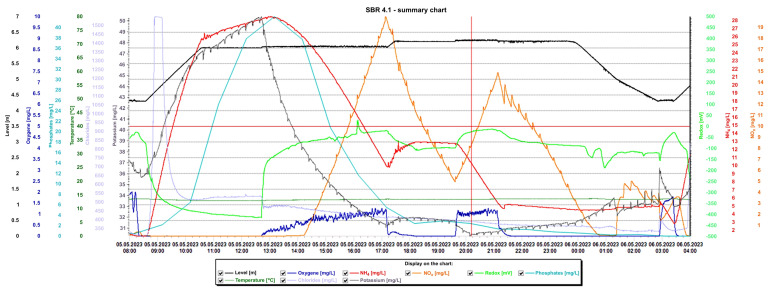
Values of selected indicators characterizing sewage treated 24 h a day in the SBR 4.1 reactor.

**Figure 16:**
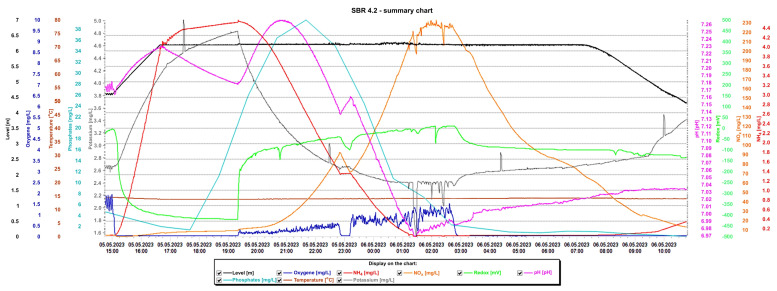
Values of selected indicators characterizing sewage treated 24 h a day in the SBR 4.2 reactor.

Detailed analysis of the graphs is presented in Figure 15 and Figure 16, presenting the values of selected indicators (sewage table level, m; O_2_ concentration, mg/L; temperature, °C; phosphates, mg/L; pH, K, mg/L, redox, mV; NO_x_, mg/L; NH_4_, mg/L) obtained continuously thanks to sensors and electrodes installed in the reactors, and showing a precise picture of the transformations taking place in the wastewater in the SBR 4.1 (Figure 15) and 4.2 reactors (Figure 16). After introducing raw sewage into the reactors, the amount of potassium, phosphates, and NH_4_ increases rapidly, and their amounts decrease over time. In the process of wastewater treatment and aeration, NO_x_ is produced, which is the sum of nitrogen oxide (NO) and nitrogen dioxide (NO_2_). NO_x_ does not include nitrous oxide (N_2_O). These oxides contribute to the formation of smog and acid rain and affect the tropospheric ozone.

NO_x_ is usually formed from the reaction of nitrogen and oxygen in the reactor as a result of the aeration of nitrogen compounds contained in the treated sewage. In the SBR 4.1 reactor, during the aeration process from 12:30 p.m. to 5:15 p.m., the oxygen concentration increased from 0 to 1.3 mg/L and the NO_x_ concentration increased from 0 to 19.97 mg/L. After turning off the aeration, the oxygen concentration dropped to approximately 0 mg/L and the NO_x_ concentration dropped to approximately 5 mg/L. After the blower was turned on again at 7:40 p.m., the oxygen concentration increased to approximately 1.35 mg/L and remained at this level until 21.15 with the simultaneous shutdown of the aeration process. During this time, the NO_x_ concentration increased again to 14.5 mg/L. After turning off the blower, the NO_x_ concentration decreased, reaching 0 mg/L at 0.45.

In the SBR 4.2 reactor, NO_x_ concentrations also varied with oxygen concentration, but the obtained values were much higher (Figure 16). In the first phase of aeration, the oxygen concentration when the blowers were turned off was 0.65 mg/L, and the NO_x_ concentration = 85 mg/L. In the second aeration phase lasting from 23.15 to 2.40 h, the maximum oxygen concentration was 1.5 mg/L and the maximum NO_x_ concentration was 233.01 mg/L, which remained at this level for approximately 1.5 h. At the same time, changes in the sewage pH were observed; as the NO_x_ concentration increased, the pH of the sewage decreased and vice versa. This means that an oxidation process was taking place. The maximum NOx concentration occured at the lowest pH values of approximately 6.98. The test results indicate a beneficial effect of the biofilm on low NO_x_ emissions, many times lower than in reactor 4.2. To confirm the results, the obtained relationships from subsequent days of observation are presented in Table 3. These are the maximum concentrations of NO_x_, NH_4_, and oxygen in the SBR 4.1 and 4.2 reactors. The results indicate that when using a mineral bed with biofilm (SBR 4.1), less oxygen is required (1.95 < 2.21 mg/L), and, therefore, less aeration of wastewater, which contributes to reducing CO_2_ emissions and energy consumption. Intensive nitrification and denitrification occur, and the amounts of nitrogen compounds in sewage are smaller. Therefore, much smaller amounts of NO_x_ are generated in the aeration process (33.87 < 195 mg/L), which reduces the greenhouse effect and smog and improves air quality.

### 3.2. Microbiological Testing

**Figure 17:**
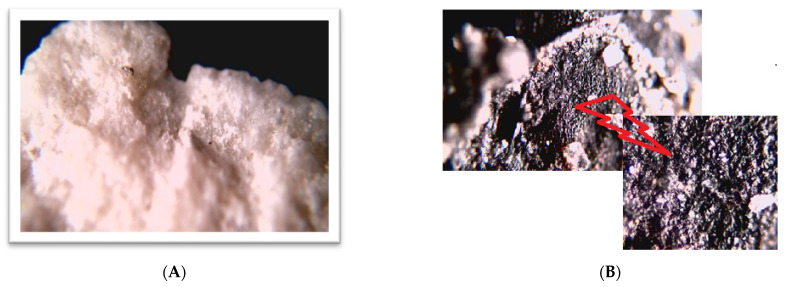
Photograph of the substrate (**A**) before testing and (**B**) after immobilization of microorganisms (with an enlarged fragment of the surface).

Microbiological tests were carried out for the biofilm formed on a mineral substrate. Figure 17 shows photographs of (A) clean substrate and (B) with biofilm. The surface of the material was covered with pores in which microorganisms developed.

**Figure 18:**
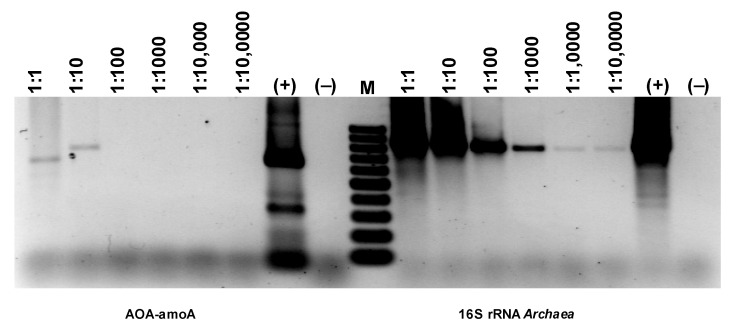

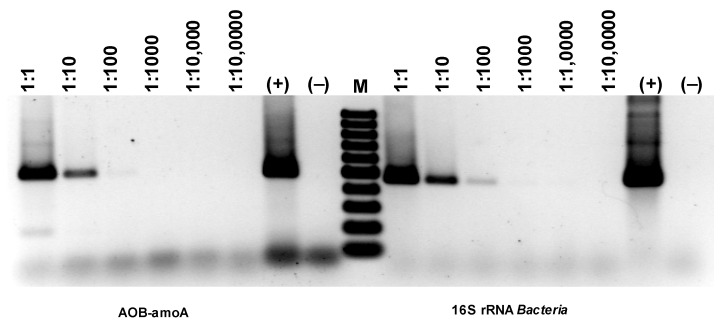
Detection of AOA and NOB based on the presence of genes specific for ammonia-oxidizing microorganisms.

Comparing the intensity of the electrophoretic bands (Figure 18), a much stronger (difference is two orders of magnitude) signal can be seen for the 16S rRNA derived from AOA than AOB (Figure 18). Since the growth dynamics of archaea are usually much lower than those of bacteria, this may mean particularly favorable conditions for developing archaea in the experimental bioreactor. However, the band for AOA amoA is extremely faint, basically at the limit of quantification via PCR. Therefore, it would follow that only a very small percentage of archaea present in the studied biofilm are microorganisms with the ammonium monooxygenase gene. Thus, the electrophoretic image shows that although the number of bacteria involved in nitrogen transformations is smaller than archaea, almost all of them have amoA activity, while archaea are the opposite. Only a few archaea among those developing in the sediment have amoA activity.

Ammonia-oxidizing *Archaea* (AOA) and bacteria (AOB) are thought to contribute differentially to the nitrification of nitrogen in wastewater [29], but the extent to which their relative abundance influences the temperature of the nitrification reaction is poorly understood. Similar relationships were obtained for nitrification occurring in soils [11].

## 4. Conclusions

Our research has shown that the process of removing nitrogen compounds is catalyzed using a mineral substrate with affinity for ammonium ions. Such a mineral substrate creates good conditions for the formation of a biofilm in which microorganisms that participate in the process of removing nitrogen compounds from sewage develop. Biofilm ensures the safe development of microorganisms, even at low sewage temperatures (<10 °C), and intense shortened nitrification and denitrification occur, similar to the Anammox process. Genetic studies (DNA/RNA) have shown that AOB and AOA bacteria and other microorganisms, such as archaea, are involved in this process. The results of genetic investigations do not determine everything, but the stable operation of the reactors clearly favors the development of archaea. On the other hand, the activity of bacteria actively oxidizing ammonia was mainly observed (the presence of the bacterial amoA gene). The process of removing nitrogen compounds requires less oxygen and, therefore, less aeration, which reduces the costs of the wastewater treatment process. Much smaller amounts of NO_x_ are generated during wastewater aeration, which is very beneficial for the environment and ongoing climate change.

## Figures and Tables

**Table 1:** Primer sequences.

Primer	Sequence	Amplicon Size (bp)
AOA amoA	F: 5′-STA ATG GTC TGG CTT AGA CG-3′	635
	R: 5′-GCG GCC ATC CAT CTG TAT GT-3′	
AOA 16S Rrna	5′-ACK GCT CAG TAA CAC GT-3′	840
	5′-YCC GGC GTT GAM TCC AAT T-3′	
AOB amoA	5′-GGG GTT TCT ACT GGT GGT-3′	491
	5′-CCC CTC KGS AAA GCC TTC TTC-3′	
AOB 16S Rrna	5′-GGAGrAAAGyAGGGGATC-3′	465
	5′-CTA GCY TTG TAG TTT CAA ACG C-3′	

**Table 2:** Physical and chemical characteristics of sampled treated sewage taken from SBR 4.1. and SBR 4.2.

Days	T °C		pH		TSS, mg/L		TN, mg/L		TP mg/L		COD, mg/L		BOD, mg/L	
	SBR 4.1.	SBR 4.2.	SBR 4.1.	SBR 4.2.	SBR 4.1.	SBR 4.2.	SBR 4.1.	SBR 4.2.	SBR 4.1.	SBR 4.2.	SBR 4.1.	SBR 4.2.	SBR 4.1.	SBR 4.2.
1	18.32	17.62	7	7.11	-	-	2.5	3.26	0.703	0.98	46.2	41.9	-	-
4	17.38	17.26	7.15	7.06	6	8	2.37	4.24	0.328	0.626	43.8	44.9	6	4
7	17.56	16.91	7.1	7.15	5	5	3.75	4.44	2.31	0.899	53.6	46.4	7	5
10	17.38	17.26	7.22	7.21	6	8	2.37	4.24	0.328	0.626	49.7	50.7	6	4
14	17.2	17.1	7.15	7.05	2	3	8.26	3.01	0.282	0.53	52.1	50.5	1	2
17	17	17.2	7.2	6.84	9	9	5.65	5.24	1.81	2.32	56.8	50.3	2	11
21	17.63	16.89	7.38	7.32	6	2	3.45	4.02	1.12	0.493	55.2	47.3	5	4
24	16.64	16.76	7.44	7.23	4	2	2.63	6.07	0.655	0.423	48.4	46	3	5
29	16.81	16.84	7.03	7.08	3	4	3.31	7.7	0.259	0.273	51.3	46.5	14	13
32	17.01	17.01	7.05	7.06	3	3	3.21	7.6	0.268	0.287	50.8	46.7	2	4
34	17.05	17.05	6.92	6.9	6	4	4.45	5.13	1.21	0.352	50.5	48.4	5	6
37	16.41	16.77	7.23	7.13	10	3	4.53	12.1	1.28	0.579	48.7	57.3	6	4
41	15.23	15.25	7.48	7.13	6	2	2.95	11.5	0.871	0.224	46.5	42.9	4	3
44	15.88	15.71	7	7.19	21	16	9	3.85	0.604	0.688	65.3	60.3	10	4
48	15.67	15	7.28	7.33	6	1	6.19	4.49	0.695	0.888	63.7	46.8	10	16
51	14.11	14.62	6.96	7.13	18	5	3.5	4.52	1.63	0.301	56.3	49.2	9	8
55	12.85	13.15	6.8	7.04	1	2	10.9	4.66	0.352	0.506	58.3	46.6	9	8
62	14.43	12.7	7.26	7.36	3	9	4.14	10.3	0.802	0.309	52.7	45	11	18
65	12.38	12.56	7.2	7.26	11	4	3.52	5.63	1.03	0.228	57.6	44.5	6	5
69	12.43	12.7	7.26	7.15	3	9	4.14	10.3	0.802	0.309	52.7	45	11	4
76	11.24	10.74	6.92	7.11	9	3	10.8	3.19	0.319	1.21	60.3	47.7	2	2
average	15.74	15.58	7.14	7.14	6.90	5.10	4.84	5.98	0.84	0.62	53.36	47.85	6.45	6.50
standard deviation	2.00	1.99	0.17	0.13	4.97	3.59	2.61	2.75	0.55	0.46	5.50	4.23	3.53	4.43

T—temperature, TSS—total suspended solids, TN—total nitrogen, TP—total phosphorus.

**Table 3:** Maximum values of selected indicators characterizing the processes taking place in reactors.

		SBR 4.1			SBR 4.2	
Days	NO_x_ mg/L	NH_4_, mg/L	O_2_, mg/L	NOx, mg/L	NH_4_, mg/L	O_2_, mg/L
5–6 May 2023	19.97	28.48	2.02	233	4.56	1.95
13–14 May 2023	44.5	31.2	2.1	181	3.7	2.13
26–27 May 2023	36.23	29.34	1.79	220	5.54	2.37
7–8 June 2023	37.98	38.92	1.79	207	4.33	2.43
10–11 June 2023	41.71	42.76	1.89	187	4.97	2.13
15–16 June 2023	22.81	43.2	2.12	142	4.28	2.23
mean	33.87	35.65	1.95	195	4.56	2.21

## Data Availability

Cracow University of Technology Faculty of EEE Project report number POIR.04.01.04-00-0039/17.
